# Survival outcomes of apalutamide as a starting treatment: impact in real-world patients with metastatic hormone sensitive prostate cancer (OASIS)

**DOI:** 10.1038/s41391-024-00929-6

**Published:** 2024-12-20

**Authors:** Benjamin L. Maughan, Yanfang Liu, Suneel Mundle, Xiayi Wang, Mehregan Nematian-Samani, Lawrence I. Karsh

**Affiliations:** 1 Huntsman Cancer Institute, University of Utah, 2000 Cir of Hope Dr, Salt Lake City, UT 84112 USA; 2 Department of Global Real-World Evidence, Janssen Pharmaceuticals LLC, 1000 U.S. Route 202, Raritan, NJ 08869 USA; 3 Global Medical Affairs, Janssen Research & Development, 743 Knoch Knolls Rd, Raritan, NJ 60565 USA; 4 Department of Janssen Data Science, 1125 Trenton Harbourton Rd, Titusville, NJ 08560 USA; 5 Medical Affairs, Solid Tumors, Janssen-Cilag GmbH, Johnson & Johnson Platz 1, 41470 Neuss, North Rhine-Westphalia Germany; 6 Advent Health Urology Denver, 850 Harvard Avenue, Denver, CO 80210 USA

**Keywords:** Cancer epidemiology, Prostate cancer

## Abstract

**Background:**

Androgen receptor pathway inhibitors (apalutamide [APA], enzalutamide [ENZ], abiraterone acetate plus prednisone [AAP]) combined with androgen-deprivation therapy (ADT) are effective life-prolonging treatment options for metastatic hormone-sensitive prostate cancer (mHSPC). We evaluated the impact of upfront therapy for mHSPC on outcomes in real-world clinical practice in the United States.

**Methods:**

This retrospective, observational cohort study used electronic healthcare records from the ConcertAI RWD 360 Prostate Cancer Dataset. All patients with newly diagnosed mHSPC from January 2018 to June 2023 were enrolled and followed-up until death, end of follow-up, or January 2024, whichever occurred first. Kaplan-Meier methods were used to estimate overall survival (OS), time to PSA50/PSA90 (50%/90% decline in PSA from baseline, respectively), time to undetectable PSA ( ≤ 0.2 ng/ml), and time to castration resistance (TTCR). Adjusted hazard ratios (aHR) were estimated using inverse probability of treatment weighted multivariate Cox proportional models adjusted for age, comorbidities, BMI, and baseline PSA.

**Results:**

4937 patients with mHSPC were included in the analysis: 315 received upfront APA + ADT, 1181 ENZ + ADT, 1760 AAP + ADT, 432 docetaxel (DTX) + ADT, and 1249 ADT alone. Percentages of patients reaching PSA50, PSA90, and undetectable PSA at 3 months were significantly higher for APA + ADT (70%/49%/44%, respectively) compared to ENZ + ADT (60%/38%/32%), AAP + ADT (59%/37%/33%) and ADT alone (32%/15%/32%). OS and TTCR were also significantly longer for APA + ADT (66%/77% respectively at 24 months) vs ENZ + ADT (55%/63%) AAP + ADT (59%/67%) and ADT alone (54%/57%). Starting treatment with APA + ADT was associated with a significantly reduced risk of death compared with ENZ + ADT (aHR, 95%CI) (0.66, 0.51–0.87), AAP + ADT (0.72, 0.55–0.94), and ADT alone (0.64, 0.49–0.84).

**Conclusions:**

Numerous patients were not treated with intensified therapies despite their increased effectiveness. First-line APA + ADT in mHSPC was associated with statistically significantly longer OS, longer TTCR, and faster and deeper PSA responses than other life-prolonging treatments in real-world clinical practice in the US.

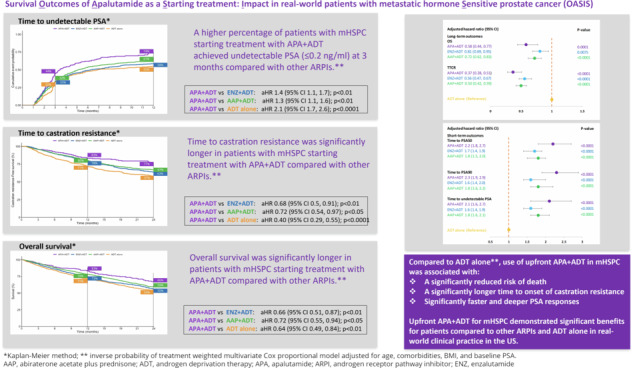

## Introduction

In 2020, approximately 209,000 men were newly diagnosed with prostate cancer in the United States (US) and more than 32,000 died [1]. Around 15% of PC cases are hormone sensitive and most patients develop castration resistance within 5 years [2]. Metastatic hormone-sensitive prostate cancer (mHSPC) is incurable, and the aim of treatment is tumor suppression to prolong survival [3].

The treatment landscape for mHSPC has evolved rapidly over the last decade. Androgen-deprivation therapy (ADT) was the mainstay of treatment for many years [4], but an increasing selection of novel hormonal therapies, notably androgen receptor pathway inhibitors (ARPIs) including apalutamide (APA), enzalutamide (ENZ), abiraterone acetate plus prednisone (AAP), and darolutamide in triple combination with ADT and docetaxel (DARO + ADT + DTX) are now available. Novel hormonal therapies combined with ADT have contributed to improved survival outcomes for patients with mHSPC [5], and are now recommended as standard of care for the treatment of mHSPC over ADT alone [6, 7].

APA is an oral ARPI that inhibits proliferation of PC cells by binding to the androgen receptor and preventing androgen receptor-mediated transcription. In the TITAN trial in mHSPC, the combination of APA + ADT reduced the risk of radiographic progression-free survival by 52% (*p* < 0.001), reduced the risk of castration resistance (CR) by 66% (*p* < 0.0001), and reduced the risk of death by 35% (*p* < 0.0001) compared to placebo+ADT [8, 9]. Treatment benefits were observed in patients across a broad range of clinical subgroups defined by tumor volume and metastases [10]. Improved survival was associated with rapid and deep declines in PSA levels to undetectable ( ≤ 0.2 ng ml) or to 90% lower than baseline (PSA90) in the first 3 months of treatment [11]. APA was approved for the treatment of patients with mHSPC in the US in 2019 [12].

The rapidly changing treatment landscape for patients with PC represents a challenge for physicians seeking to achieve optimal clinical outcomes for their patients. Real-world evidence can help inform clinical decision-making with respect to treatment selection and sequencing. We used real-world data to compare the impact of the starting treatment on short-term and long-term clinical outcomes in mHSPC in the US.

## Subjects and methods

### Data source

ConcertAI integrates and curates data from electronic health records (EHRs) for a population of over four million oncology patients through their network of 900 geographically distributed US oncology clinic sites. It receives all patient EHRs as structured and unstructured data, and contains laboratory and imaging results, physician notes, progress notes, surgical reports, and other documents collected as part of routine inpatient and outpatient care. Variables such as biomarkers can be retrieved from unstructured documents. ConcertAI RWD360 is a research grade data source in which critical clinical data such as staging, PSA values and CR status are captured.

### Study design and population

This retrospective observational cohort study evaluated the impact of the starting treatment on clinical outcomes in patients with mHSPC. The study population comprised all individuals aged ≥18 years with a confirmed diagnosis of mHSPC in the database who received a relevant upfront treatment for PC ( ≥ 1 cycle of any ARPI, ADT, combined androgen blockade, DTX, radiotherapy, or prostatectomy). This analysis focused on patients who started treatment with one of the following: APA + ADT, ENZ + ADT, AAP + ADT, DARO + ADT + DTX, DTX + ADT, or ADT alone. The index date was date of the first treatment received for mHSPC during the study period.

Patients were identified by the International Classification of Disease (version 10) (ICD10) code C61 in the database, were required to have a diagnosis or evidence of HSPC during the study period (defined in the supplement), and at least six months of continuous enrollment in database prior to the index date. Patients were excluded if they had a diagnosis of any other malignancy (other than non-melanoma skin cancer or bladder cancer treated only with transurethral resection) prior to being diagnosed with PC. Hormone sensitivity was confirmed by meeting at least one of the following criteria (provided in full in the Supplement):

- At least one diagnosis code indicating hormone sensitive malignancy (ICD10 code Z19.1) status within 12 months prior to/on the index date,
- No claims/diagnosis of CR prior to the index date and no claim for ADT for 18 months prior to the index date,
- At least one claim for surgical castration and at least two PSA test results after surgical castration and within 12 months prior to/on the index date, without evidence of biochemical progression,
- Medical castration (at least 90 days of continuous ADT) prior to the index date and at least two PSA test results within the same ADT episode, and within 12 months prior to/on the index date, without evidence of biochemical progression.

Patient were enrolled from 1 January 2018 to 30 June 2023 and were followed up for death, loss to follow-up, or study end on 18 January 2024, whichever occurred first.

### Ethics approval and consent to participate

All data were de-identified by ConcertAI before disclosure; therefore, ethical review and patient consent were not required. The study was conducted according to all applicable guidelines and regulations.

### Exposure and outcomes

Treatment groups were defined by starting treatment. Short-term outcomes included undetectable PSA at 3 months and 12 months, time to ≥50% and ≥90% decline in PSA (PSA50/PSA90), and time to undetectable PSA (≤0.2 ng/ml), from the last baseline value. Long-term outcomes were overall survival (OS) and time to onset of CR (TTCR).

### Statistical analysis

Descriptive statistics were used to summarize study variables. Continuous variables were summarized by mean, and standard deviation (SD), median, quartiles (Q1, Q3), and categorical variables by frequencies and percentages. Time-to-event variables (TTCR, time to death and time to PSA reduction) were summarized using the Kaplan-Meier method. Adjusted hazard ratios (aHR) were estimated for each treatment group compared to ADT alone. All HRs were presented with two-sided 95% Wald confidence intervals (CI). Pair-wise comparisons were performed for all outcomes for APA + ADT compared to each treatment group using Inverse Probability of Treatment Weighted (IPTW) multivariate Cox proportional hazard models adjusted for age, Charlson Comorbidity index (CCI) score, body mass index, and baseline PSA. The IPTW method weights each group according to the inverse of the propensity score to account for the different sample size of each treatment group [13].

#### Sample size

Based on the TITAN trial, the mortality rate was 0.19 and the HR for death was 0.67 for APA + ADT compared with ADT alone [8]. A minimum combined sample size of 1031 for APA + ADT and ADT alone was needed to detect a HR of at least 0.67 with 80% statistical power and a significance level of 0.05. For the TTCR analysis, the disease progression rate was 0.35 and the HR was 0.48 for APA + ADT compared with ADT alone. A minimum combined sample size of 172 for APA + ADT and ADT alone was needed to detect a HR of at least 0.48 with 80% statistical power and a significance level of 0.05. For PSA outcomes, 66% of patients achieved PSA90, and the HR was 1.49 between groups receiving APA compared to ENZ [14]. A minimum sample size of 300 for the APA + ADT and ENZ + ADT groups was needed for the PSA90 analysis to detect a HR of at least 1.49 with 80% statistical power and a significance level of 0.05. Based on this feasibility assessment the sample size in each of the cohorts was sufficient to detect differences between APA + ADT and other groups.

## Results

### Patient population

There were 9136 eligible patients with a new diagnosis of mHSPC during the study period (Fig. S1), of which 4937 were included in the comparative analysis: 315 who received upfront treatment with APA + ADT, 1181 treated with ENZ + ADT, 1760 treated with AAP + ADT, 432 treated with DTX + ADT, and 1249 treated with ADT alone (Table 1). As only 62 patients received upfront DARO + ADT + DTX with limited follow-up, this group was not included in the analysis.

**Table 1:** Demographic and clinical characteristics of patients at baseline, by starting treatment.

	APA + ADT *N* = 315	ENZ + ADT *N* = 1181	AAP + ADT *N* = 1760	DTX + ADT *N* = 432	ADT alone *N* = 1249
**Age**, mean (SD)	73.08 (8.68)	74.38 (8.68)	73.63 (8.82)	68.99 (9.00)	74.20 (9.08)
18–54	2 (0.6%)	27 (2.3%)	35 (2.0%)	22 (5.1%)	29 (2.3%)
55–64	61 (19.4%)	139 (11.8%)	261 (14.8%)	106 (24.5%)	177 (14.2%)
65–74	99 (31.4%)	394 (33.4%)	610 (34.7%)	184 (42.6%)	379 (30.3%)
75–84	122 (38.7%)	467 (39.5%)	670 (38.1%)	106 (24.5%)	508 (40.7%)
>85	31 (9.8%)	154 (13.0%)	184 (10.5%)	14 (3.2%)	156 (12.5%)
**BMI**, mean (SD)	27.83 (4.89)	27.72 (4.91)	27.71 (4.47)	27.57 (4.99)	27.91 (4.83)
Underweight ( < 18.5 kg/m^2^)	0	0	0	0	0
Normal weight (18.5 to <25 kg/m^2^)	56 (31.3%)	200 (29.3%)	313 (28.1%)	83 (31.3%)	251 (29.8%)
Overweight (25.0 to <30.0 kg/m^2^)	71 (39.7%)	285 (41.7%)	489 (44.0%)	106 (40.0%)	311 (37.0%)
Obese ( ≥ 30.0 kg/m^2^)	52 (29.1%)	198 (29.0%)	310 (27.9%)	76 (28.7%)	279 (33.2%)
Unknown	136 (43.2%)	498 (42.2%)	648 (36.8%)	167 (38.7%)	408 (32.7%)
**Site of metastases**, *n* (%)					
Bone	188 (88.7%)	738 (89.7%)	1056 (87.6%)	251 (75.8%)	702 (81.6%)
Nodal	15 (7.1%)	45 (5.5%)	88 (7.3%)	24 (7.3%)	85 (9.9%)
Visceral	9 (4.2%)	40 (4.9%)	61 (5.1%)	56 (16.9%)	73 (8.5%)
Unknown	103 (32.7%)	358 (30.3%)	555 (31.5%)	101 (23.4%)	389 (31.1%)
**Baseline PSA (ng/dL)**, median (Q1, Q3)	6.1 (1.0, 34.1)	10.7 (1.5, 47.0)	11.0 (1.3, 51.3)	19.8 (3.2, 96.5)	1.7 (0.1, 20.4)
**Mean** (SD)	41.28 (86.65)	50.51 (92.64)	53.47 (94.82)	74.61 (112.43)	34.50 (81.92)
**Baseline ECOG PS**, *n* (%)					
Not available	136 (43.2%)	508 (43.0%)	861 (48.9%)	172 (39.8%)	599 (48.0%)
0–1	144 (80.4%)	528 (78.5%)	720 (80.1%)	211 (81.2%)	519 (79.8%)
2–3	34 (19.0%)	137 (20.4%)	175 (19.5%)	47 (18.1%)	126 (19.4%)
4+	1 (0.6%)	8 (1.2%)	4 (0.4%)	2 (0.8%)	5 (0.8%)
**CCI**, median (Q1, Q3)	0.0 (0.0,1.0)	0.0 (0.0, 1.0)	0.0 (0.0, 1.0)	0.0 (0.0, 1.0)	0.0 (0.0, 1.0)
**CCI score**, *n* (%)					
**0**	210 (66.7%)	823 (69.7%)	1187 (67.4%)	294 (68.1%)	796 (63.7%)
**1**	49 (15.6%)	150 (12.7%)	290 (16.5%)	78 (18.1%)	204 (16.3%)
**2**	35 (11.1%)	100 (8.5%)	160 (9.1%)	35 (8.1%)	132 (10.6%)
**3**	11 (3.5%)	52 (4.4%)	59 (3.4%)	12 (2.8%)	57 (4.6%)
**4**	5 (1.6%)	26 (2.2%)	33 (1.9%)	4 (0.9%)	30 (2.4%)
**5** +	5 (1.6%)	30 (2.5%)	31 (1.8%)	9 (2.1%)	30 (2.4%)
**Comorbiditie**s, *n* (%)					
AIDS	0 (0.0%)	2 (0.2%)	6 (0.3%)	0 (0.0%)	3 (0.2%)
Acute myocardial infarction	7 (2.2%)	14 (1.2%)	12 (0.7%)	6 (1.4%)	18 (1.4%)
Cerebrovascular disease	12 (3.8%)	41 (3.5%)	64 (3.6%)	13 (3.0%)	58 (4.6%)
COPD	9 (2.9%)	63 (5.3%)	121 (6.9%)	30 (6.9%)	87 (7.0%)
Congestive heart failure	17 (5.4%)	67 (5.7%)	115 (6.5%)	24 (5.6%)	83 (6.6%)
Dementia	5 (1.6%)	19 (1.6%)	19 (1.1%)	0 (0.0%)	16 (1.3%)
Diabetes	37 (11.7%)	125 (10.6%)	155 (8.8%)	55 (12.7%)	138 (11.0%)
Diabetes with complications	10 (3.2%)	57 (4.8%)	54 (3.1%)	14 (3.2%)	48 (3.8%)
History of myocardial infarction	4 (1.3%)	17 (1.4%)	21 (1.2%)	6 (1.4%)	17 (1.4%)
Mild liver disease	12 (3.8%)	28 (2.4%)	38 (2.2%)	12 (2.8%)	42 (3.4%)
Moderate/severe liver disease	0 (0.0%)	2 (0.2%)	3 (0.2%)	1 (0.2%)	5 (0.4%)
Paralysis	4 (1.3%)	6 (0.5%)	14 (0.8%)	1 (0.2%)	7 (0.6%)
Peptic ulcer disease	3 (1.0%)	8 (0.7%)	7 (0.4%)	3 (0.7%)	9 (0.7%)
Peripheral vascular disease	13 (4.1%)	56 (4.7%)	87 (4.9%)	25 (5.8%)	80 (6.4%)
Renal disease	28 (8.9%)	101 (8.6%)	142 (8.1%)	21 (4.9%)	127 (10.2%)
Rheumatologic disease	2 (0.6%)	5 (0.4%)	17 (1.0%)	5 (1.2%)	8 (0.6%)
**Treatment duration (months)**, median (Q1, Q3)	13.4 (6.4, 24.4)	12.3 (5.7, 23.3)	14.1 (6.5, 25.5)	7.9 (4.1, 15.7)	10.3 (3.7, 19.2)

*AIDS* acquired immune deficiency syndrome, *AAP* abiraterone acetate plus prednisone, *ADT* androgen deprivation therapy, *APA* apalutamide, *BMI* body mass index, *CCI* Charlson Comorbidity Index, *COPD* chronic obstructive pulmonary disease, *DTX* docetaxel, *ENZ* enzalutamide, *SD* standard deviation, *Q1, Q3* first and third quartiles.

The mean age of patients was similar in each group (range of means 69.0 to 74.4 years). Compared to patients who received an ARPI + ADT or ADT alone, patients who received upfront DTX + ADT tended to have more visceral metastases (16.9% versus 4.2–8.5%). CCI scores were distributed similarly between groups. The most frequently occurring comorbidities in each treatment group were diabetes (8.8 to 12.7% of patients), congestive heart failure (5.4% to 6.6%), and renal disease (4.9 to 10.2%).

### Short-term outcomes

#### Time to PSA50 and PSA90

**Fig. 1:**
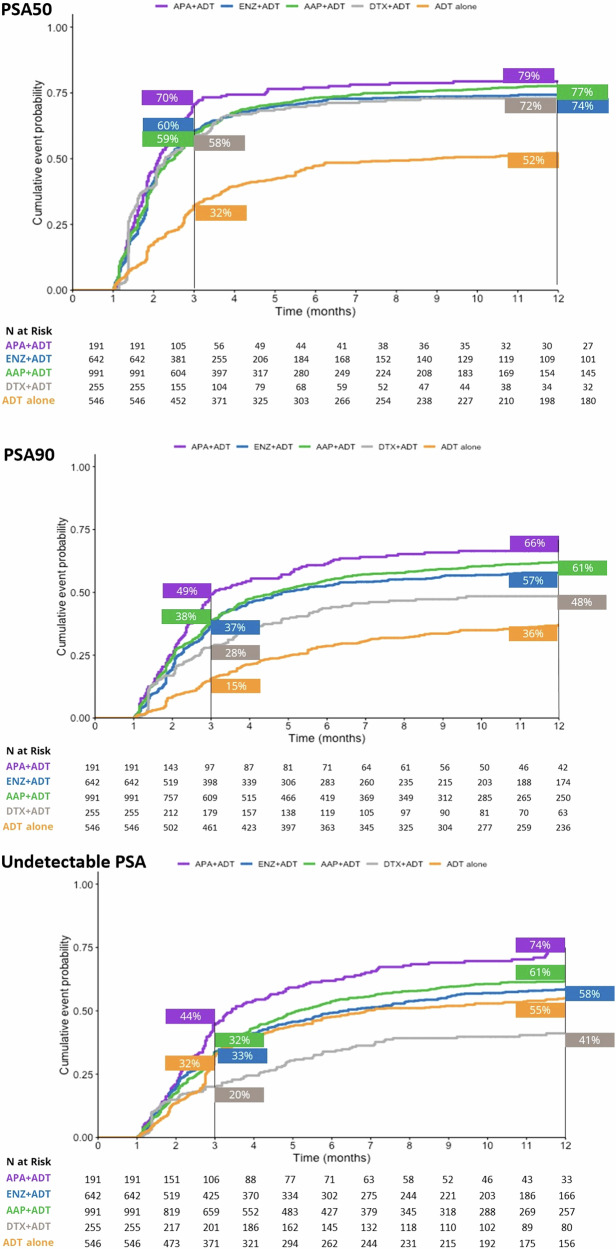
Time to PSA50, PSA90 and undetectable PSA ( < 0.2 ng/ml) by starting treatment in patients with mHSPC – Kaplan-Meier method. AAP abiraterone acetate plus prednisone, ADT androgen deprivation therapy, APA apalutamide, DTX docetaxel, ENZ enzalutamide, mHSCP metastatic hormone-sensitive prostate cancer, PSA50/PSA90 50%/90% decline in PSA from baseline, Undetectable PSA, ≤0.2 ng/ml.

**Fig. 2:**
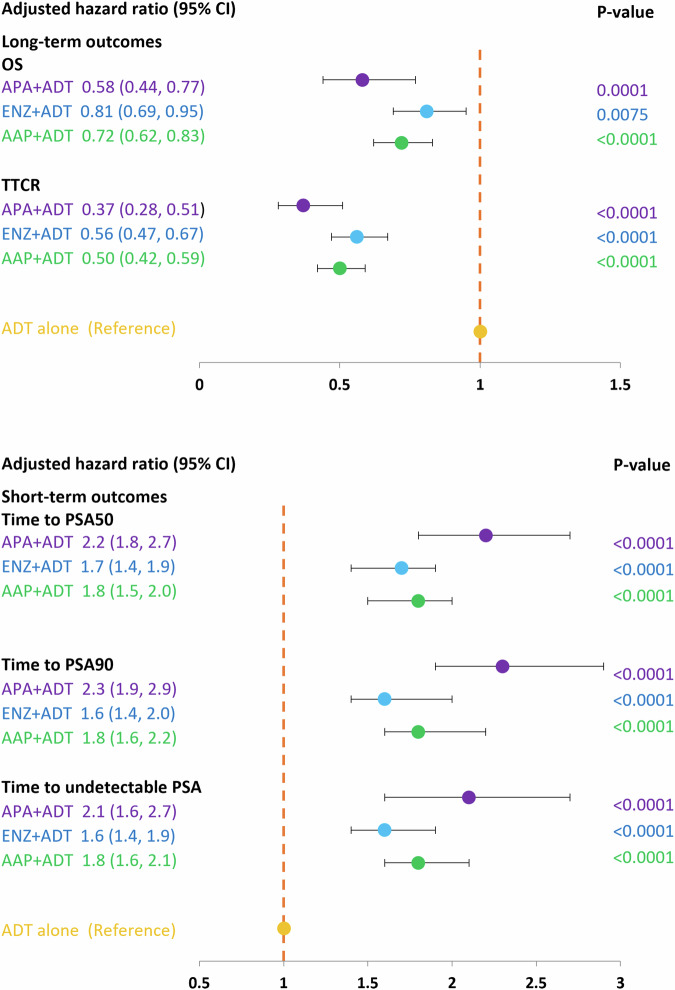
Multivariate Cox regression model* results for hazard ratios by starting treatment group in patients with mHSPC; ARPIs + ADT as compared to ADT alone. AAP abiraterone acetate plus prednisone, ADT androgen deprivation therapy, APA apalutamide, DTX docetaxel, ENZ enzalutamide, mHSCP metastatic hormone-sensitive prostate cancer, OS overall survival, PSA50/PSA90 50%/90% decline in PSA from baseline, TTCR time to castration resistance, Undetectable PSA, ≤0.2 ng/ml. *Adjusted for age, comorbidities, BMI and baseline PSA level.

At the 3-month timepoint, 70% of patients who initiated APA + ADT had reached PSA50 versus 60% in the ENZ + ADT, 59% in the AAP + ADT group, 58% in the DTX + ADT group, and 32% in the ADT alone group (Fig. 1). At month 12, 79% of patients in the APA + ADT group had reached PSA50 versus 77%, 74%, 72%, and 52%, respectively. The median time to PSA50 was 2.13 months in the APA + ADT group, 2.33 months in the ENZ + ADT group, 2.52 months in the AAP + ADT group, 2.30 months in the DTX + ADT group, and 8.98 months in the ADT alone group (Table S1). Adjusted HRs for the likelihood of reaching PSA50 at any time were significantly higher in all groups compared to ADT alone (Fig. 2). The likelihood of reaching PSA50 was significantly higher for APA + ADT compared with ENZ + ADT (aHR 1.4 [95% CI 1.2–1.7]), AAP + ADT (aHR 1.3 [95% CI 1.1–1.6]), DTX + ADT (aHR 1.7 [95% CI 1.4–2.1]), and ADT alone (aHR 2.4 [95% CI 1.9–2.9]) (Table 2).

**Table 2:** Pair-wise comparisons assessing outcomes in patients starting on APA + ADT compared with other treatments—adjusted^a^ multivariate Cox regression using the IPTW method.

Upfront treatment	Comparison	Adjusted hazard ratio (95% CI)^b^	*p* value
**PSA50 at any time**			
APA + ADT	vs ENZ + ADT	1.4 (1.2, 1.7)	<0.001
APA + ADT	vs AAP + ADT	1.3 (1.1, 1.6)	<0.01
APA + ADT	vs DTX + ADT	1.7 (1.4, 2.1)	<0.0001
APA + ADT	vs ADT alone	2.4 (1.9, 2.9)	<0.0001
**PSA90 at any time**			
APA + ADT	vs ENZ + ADT	1.5 (1.2, 1.8)	<0.001
APA + ADT	vs AAP + ADT	1.3 (1.1, 1.6)	<0.01
APA + ADT	vs DTX + ADT	2.5 (1.9, 3.3)	<0.0001
APA + ADT	vs ADT alone	2.4 (1.9, 3.1)	<0.0001
**Undetectable PSA at any time**			
APA + ADT	vs ENZ + ADT	1.4 (1.1, 1.7)	<0.01
APA + ADT	vs AAP + ADT	1.3 (1.1, 1.6)	<0.01
APA + ADT	vs DTX + ADT	2.0 (1.5, 2.6)	<0.0001
APA + ADT	vs ADT alone	2.1 (1.7, 2.6)	<0.0001
**Overall survival**			
APA + ADT	vs ENZ + ADT	0.66 (0.51, 0.87)	<0.01
APA + ADT	vs AAP + ADT	0.72 (0.55, 0.94)	<0.05
APA + ADT	vs DTX + ADT	0.38 (0.28, 0.52)	<0.0001
APA + ADT	vs ADT alone	0.64 (0.49, 0.84)	<0.01
**Castration-free surviva**			
APA + ADT	vs ENZ + ADT	0.68 (0.50, 0.91)	<0.01
APA + ADT	vs AAP + ADT	0.72 (0.51, 0.97)	<0.05
APA + ADT	vs DTX + ADT	0.32 (0.23, 0.44)	<0.01
APA + ADT	vs ADT alone	0.40 (0.29, 0.55)	<0.0001

*AAP* abiraterone acetate plus prednisone, *ADT* androgen deprivation therapy, *APA* apalutamide, *CI* confidence interval, *DTX* docetaxel, *ENZ* enzalutamide, *IPTW* inverse probability of treatment weighted, *NR* not reached.

^a^Adjusted for age, comorbidities, BMI and baseline PSA level.

^b^Small differences may exist between aHRs observed between this pairwise analysis and the multivariate analysis presented in Fig. 2 as a result of the analysis methods.

Similar findings were observed for PSA90. At 3 months, 49% of patients initiated on APA + ADT had reached PSA90 versus 38% in the ENZ + ADT group, 37% in the AAP + ADT group, 28% in the DTX + ADT group, and 15% in the ADT alone group (Fig. 1). At month 12, the percentages were 66%, 61%, 57%, 48%, and 36%, in the respective groups, and the median time to PSA90 was 3.08 months, 4.82 months, 4.62 months, 13.54 months, and was not reached for ADT alone (Table S1). Adjusted HRs for the likelihood of reaching PSA90 at any time were significantly higher in the ARPI + ADT groups compared to ADT alone (Fig. 2). The likelihood of reaching PSA90 was significantly higher for APA + ADT compared with ENZ + ADT (aHR 1.5 [95% CI 1.2–1.8]), AAP + ADT (aHR 1.3 [95% CI 1.1–1.6]), DXT + ADT (aHR 2.5 [95% CI 1.9–3.3] and ADT alone (aHR 2.4 [95% CI 1.9–3.1]) (Table 2).

#### Time to undetectable PSA ( ≤ 0.2 ng/ml)

At 3 months, 44% of patients who initiated APA + ADT had undetectable PSA versus 32% in the ENZ + ADT group, 33% in the AAP + ADT group, 32% in the DTX + ADT group, and 20% in the ADT alone group (Fig. 1). At month 12, the percentages were 74%, 61%, 58%, 55, and 41%, in the respective groups, and the median time to undetectable PSA was 3.44 months, 6.30 months, 5.15 months, 22.62 months, and 6.89 months (Table S1). Adjusted HRs for the likelihood of reaching undetectable PSA were significantly higher in the ARPI + ADT groups compared to ADT alone (Fig. 2). The likelihood of reaching undetectable PSA was significantly higher for APA + ADT compared with ENZ + ADT (aHR 1.4 [95% CI 1.1–1.7]), AAP + ADT (aHR 1.3 [95% CI 1.1–1.6]), DTX + ADT (aHR 2.0 [95% CI 1.5–2.6]) and ADT alone (aHR 2.1 [95% CI 1.7–2.6]) (Table 2).

### Long-term outcomes

#### Overall survival

**Fig. 3:**
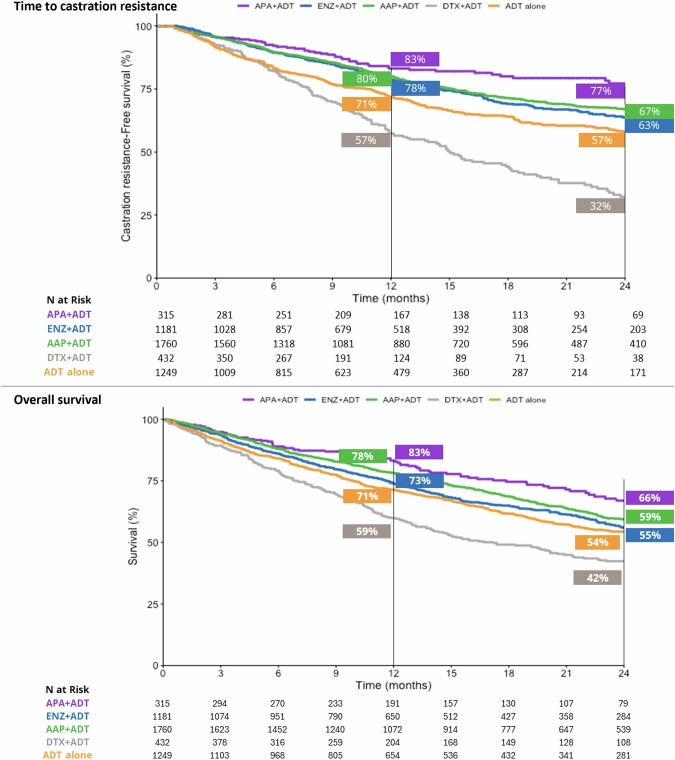
Time to castration-resistance and overall survival by starting treatment in mHSPC – Kaplan-Meier method. AAP abiraterone acetate plus prednisone, ADT androgen deprivation therapy, APA apalutamide, DTX docetaxel, ENZ enzalutamide, mHSCP metastatic hormone-sensitive prostate cancer.

At 24 months, 66% of patients who initiated APA + ADT were alive, versus 55% in the ENZ + ADT group, 59% in the AAP + ADT group, 42% in the DTX + ADT group, and 54% in the ADT alone group (Fig. 3). Median OS was not reached in the APA + ADT group, and was 28.56 months, 34.52 months, 17.18 months, and 26.79 months in the respective groups (Table S1). There was a significantly reduced risk of death in all ARPI + ADT groups compared to ADT alone. The risk of death was significantly higher in the DTX + ADT group than the ADT alone group (Fig. 2). Adjusted HRs [95% CI] for the risk of death were significantly lower in the APA + ADT group compared with ENZ + ADT (0.66 [0.51–0.87]), AAP + ADT (0.72 [0.55–0.94]), DTX + ADT (0.38 [0.28–0.52]) and ADT alone (0.64 [0.49–0.84]) (Table 2).

Approximately 40% of patients in each group had a missing baseline ECOG PS. We evaluated OS in patients with known ECOG PS (0–1 or ≥2) versus those with an unknown score. OS in patients with an unknown score strongly resembled that in patients with ECOG PS 0–1 (p = 0.86) (Fig. S2), suggesting that ECOG PS was not an important confounding factor in our study.

#### Time to castration resistance

At the 12-month timepoint, 83% of patients who started treatment with APA + ADT continued to be hormone sensitive versus 78% in the ENZ + ADT group, 80% in the AAP group, 57% in the DTX + ADT group, and 72% in the ADT alone group (Fig. 3). At 24 months the percentages were 77%, 63%, 67%, 32%, and 57%, respectively. The median TTCR was not reached in the APA + ADT group, and was 43.64 months in the ENZ + ADT group, 54.39 months in the AAP group, 15.05 in the DTX + ADT group, and 34.52 months in the ADT alone group (Table S1). The TTCR was significantly longer in the ARPI + ADT groups compared to ADT alone (Fig. 2). Adjusted HRs [95% CI] showed significantly longer TTCR when the starting treatment was with APA + ADT compared with ENZ + ADT (0.68 [0.50–0.91]), AAP + ADT (0.72 [0.54–0.97]), DTX + ADT (0.32 [0.23–0.44]), and ADT alone (0.40 [0.29–0.55]) (Table 2).

## Discussion

This real-world study using EHR data confirms that ARPIs offer clinical benefits to patients with mHSPC in terms of achieving rapid declines in PSA, delaying the development of CR, and prolonging survival compared to ADT alone. Among the ARPIs assessed in this study, use of APA + ADT as starting treatment had the greatest positive impact on all short-term and long-term outcomes studied. Additionally, APA + ADT was associated with faster PSA responses and a statistically significantly lower risk of death than other treatments.

PSA responses are considered prognostic indicators for disease progression and survival in metastatic PC [15]. Our retrospective real-world study supports findings described in clinical trial data and real-world case series showing associations between PSA kinetics and long-term clinical outcomes in patients treated with ARPIs+ADT [11, 16–18]. Our study demonstrates that PSA response rates are earlier and deeper when APA + ADT is used upfront for mHSPC compared to upfront ENZ + ADT and AAP + ADT, which likely explains the longer OS and delayed onset of CR that we observed. Further analyses of the kinetics of the PSA response and clinical outcome after APA + ADT in the real-world setting are ongoing.

Despite the availability of highly efficacious ARPI treatments, a high number of patients in this cohort did not receive intensified treatment, even though ADT alone is no longer recommended as starting therapy in patients with mHSPC and adequate life expectancy [6, 7]. The clinical reasoning underlying treatment selection is not usually captured in EHRs; however, patients with mHSPC initiated on ADT alone in our study were of a similar age and had a similar distribution of CCI scores and comorbidities as patients initiated on ARPIs. This could suggest underutilization of ARPIs in the patient population, and/or the presence of other clinical considerations that influence treatment selection. Of note, the use of ADT alone in our study is lower than previous reports from other real-world datasets (Medicare and Optum health insurance claims database) which estimated that in 2018–2019, 40–50% of men with mHSPC continued to receive first-line treatment with ADT alone [19–21]. This difference could reflect differences in the datasets (CONCERTAI is an oncology database based on medical records, whereas Medicare and Optum are claims-based from oncology, pharmacy, and community sources), different prescribing practices between oncologists and urologists, or a real decrease in the use of ADT alone over time. Nevertheless, the rate of ADT monotherapy as reported in our study is still, arguably, higher than ideal.

There were 432 patients in the database who received upfront treatment with DTX + ADT. These were a heterogenous group of patients with respect to the number of treatment cycles received and subsequent therapies. OS was very low in patients who received fewer than 6 cycles of DTX + ADT (8% at 24 months) versus 56% in patients who received at least six cycles (Fig. S3). While we included both populations in the comparative analyses as representative of real-world practice, the results need to be interpreted in light of the disparate outcomes experienced within this group of patients.

Strengths of the study include the use of a research grade repository of data collected in real-world practice allowing investigation of a large patient population with prolonged follow-up, allowing a more nuanced comparative evaluation of drug efficacy than possible in clinical trials.

Potential limitations are that mHSPC is not defined under ICD10 and while we identified patients with mHSPC using an algorithm, it is possible that some may have been excluded from the study sample if information to confidently assess their hormone sensitivity status was insufficient. The number of patients who had received DARO + ADT + DTX was low and the duration of follow-up too short to provide meaningful results. Residual confounding from unmeasured confounders is a concern. For example, we could not account for CHAARTED disease volume, Gleason score, or the presence of known high-risk genomic factors. Evaluation of additional drug combinations will be included in future evaluations as the sample size allows. ConcertAI RWD360 is an oncology database and reporting of unrelated comorbidities may not be complete. Therefore, comorbidities may have been under-reported in this database and need further evaluation in other studies.

In conclusion, the results from this real-world study confirm clinical trial findings showing clinical benefits with the use of ARPIs+ADT over ADT alone. In addition, the study expands our understanding of optimal starting treatment for mHSPC, showing the greatest benefits for patients when the starting treatment is APA + ADT. Use of upfront APA + ADT in mHSPC was associated with the fastest time to PSA50, time to PSA90 and time to undetectable PSA, the longest TTCR, and the highest reduction in the risk of death compared with the other life-prolonging therapies evaluated in this study. In view of continuing high rates of ADT alone, further research to understand patient and physician determinants of treatment selection could help to optimize treatment choice and outcomes for patients with mHSPC.

## Supplementary information

Supplemental material;

## Data Availability

Data were obtained under license and their deposition in a public repository is prohibited by ConcertAI. Interested researchers can obtain the data through formal application to the ConcertAI.

## Code availability

Computer codes used in the analysis are provided at (web address will be provided if the paper is accepted for publication).
