# Supplementary material for: Survival outcomes of apalutamide as a starting treatment: impact in real-world patients with metastatic hormone sensitive prostate cancer (OASIS)
Source: Prostate Cancer Prostatic Dis. 2024 Dec 20;28(4):865–73. doi: 10.1038/s41391-024-00929-6 (PMC12643925; doi:10.1038/s41391-024-00929-6)
Supplement: Supplementary file 1 — Supplemental material; [file 41391_2024_929_MOESM1_ESM.docx]

**Survival Outcomes of Apalutamide as a Starting treatment: Impact in real-world patients with metastatic hormone Sensitive prostate cancer (OASIS)**

Benjamin L. Maughan, Yanfang Liu, Suneel Mundle, Xiayi Wang, Mehregan Nematian-Samani, Lawrence I. Karsh

**SUPPLEMENT**

**Criteria for hormone sensitivity:**

Meeting ≥1 of the following criteria to confirm hormone sensitivity:

- ≥1 diagnosis code indicating hormone sensitive malignancy (Z19.1) status within 12 months prior to or on the index date.
- Hormone/castration naïve, defined as no claims/diagnosis of castration resistance prior to the index date (using all available data from the beginning of data stream) and no claims for ADT in the 18 months pre-index (only applicable to patients with ≥18 months continuous enrollment prior to the index date; if patients have between 12- and 18-months continuous enrollment prior to the index date, 12 month data will be utilized).
- ≥1 claim for surgical castration (i.e., bilateral orchiectomy or two unilateral orchiectomies [including one on right side and one on left side]) at any time point prior to the index date (using all available data from the beginning of data stream), AND ≥2 or PSA test results (including one nadir and one post-nadir) after the surgical castration and within 12 months prior to or on the index date. Without evidence of biomedical progression –PSA levels of ≥2 ng/mL and ≥25% increase from nadir.
- Medical castration (i.e., ≥1 episode of ≥ 90 days continuous ADT) prior to the index date AND ≥2 PSA test results (including one nadir and one post-nadir) within the same continuous ADT episode (≥ 90 days continuous ADT) and within 12 months prior to or on the index date. Without evidence of biomedical progression – PSA levels of ≥2 ng/mL and ≥25% increase from nadir.

**Table S1**  Median time to progression for PSA and survival outcomes by starting treatment

| **Upfront treatment** | **N** | **N events** | **Median**  **progression (95% CI)** | **% progression at 6 months (95% CI)** | **% progression at 12 months (95% CI)** | **% progression at 24 months (95% CI)** | |
| --- | --- | --- | --- | --- | --- | --- | --- |
| **Time to PSA50** | | | |  |  |  |  |
| APA+ADT | 191 | 153 | 2.13 (1.87, 2.46) | 22.97 (17.71, 29.8) | 20.62 (15.57, 27.31) | – | |
| ENZ+ADT | 642 | 480 | 2.33 (2.10, 2.59) | 28.37 (25.07, 32.1) | 25.72 (22.49, 29.41) | – | |
| AAP+ADT | 991 | 769 | 2.52 (2.26, 2.69) | 26.81 (24.18, 29.74) | 22.40 (19.87, 25.25) | – | |
| DTX+ADT | 255 | 184 | 2.30 (2.07, 2.75) | 29.82 (24.6, 36.16) | 27.08 (21.94, 33.43) |  | |
| ADT alone | 546 | 290 | 8.98 (5.74, 22.75) | 52.74 (48.68, 57.13) | 47.90 (43.79, 52.39) | – | |
| **Time to PSA90** |  |  |  |  |  |  | |
| APA+ADT | 191 | 129 | 3.08 (2.75, 5.08) | 38.65 (32.32, 46.23) | 33.54 (27.42, 41.04) | – | |
| ENZ+ADT | 642 | 379 | 4.82 (4.07, 6.75) | 47.33 (43.60, 51.38) | 42.19 (38.45, 46.3) | – | |
| AAP+ADT | 991 | 622 | 4.62 (3.90, 5.51) | 45.04 (42.03, 48.27) | 38.06 (35.06, 41.33) | – | |
| DTX+ADT | 255 | 121 | 13.54 (6.1, NR) | 56.32 (50.42, 62.92) | 51.54 (45.48, 58.41) |  | |
| ADT alone | 546 | 207 | NR (37.38, NR) | 71.31 (67.59, 75.24) | 63.01 (58.95, 67.36) | – | |
| **Time to undetectable PSA (≤ 0.2 ng/ml)** | | | |  |  |  | |
| APA+ADT | 191 | 142 | 3.44 (2.95, 4.82) | 38.15 (31.84, 45.71) | 25.39 (19.68, 32.75) | – | |
| ENZ+ADT | 642 | 374 | 6.30 (5.31, 8.23) | 50.85 (47.10, 54.89) | 41.37 (37.56, 45.55) | – | |
| AAP+ADT | 991 | 633 | 5.15 (4.62, 5.97) | 46.22 (43.20, 49.47) | 38.39 (35.37, 41.66) | – | |
| DTX+ADT | 255 | 106 | 22.62 (16.49, NR) | 66.08 (60.40, 72.28) | 58.90 (52.84, 65.66) |  | |
| ADT alone | 546 | 302 | 6.89 (5.51, 11.8) | 52.38 (48.33, 56.77) | 44.94 (40.81, 49.49) | – | |
| **Time to castration resistance** | | |  |  |  |  | |
| APA+ADT | 315 | 61 | NR (36.1, NR) | 92.13 (89.08, 95.27) | 83.13 (78.64, 87.88) | 77.21 (71.5, 83.38) | |
| ENZ+ADT | 1181 | 295 | 43.64 (35.57, NR) | 89.48 (87.63, 91.36) | 78.67 (76.0, 81.43) | 63.79 (60.01, 67.8) | |
| AAP+ADT | 1760 | 469 | 54.39 (43.64, NR) | 89.61 (88.12, 91.12) | 80.06 (77.99, 82.2) | 67.02 (64.25, 69.9) | |
| DTX+ADT | 432 | 182 | 15.05 (12.82, 18.2) | 82.11 (78.27, 86.15) | 57.40 (51.90, 63.48) | 32.19 (26.03, 39.8) | |
| ADT alone | 1249 | 374 | 34.52 (26.69, 49.38) | 83.29 (81.10, 85.54) | 71.76 (68.9, 74.74) | 57.88 (54.1, 61.93) | |
| **Overall survival** | | | |  |  |  |  |
| APA+ADT | 315 | 88 | NR (32.98, NR) | 88.99 (85.57, 92.55) | 83.26 (79.05, 87.69) | 66.98 (60.77, 73.81) | |
| ENZ+ADT | 1181 | 467 | 28.56 (25.34, 33.8) | 86.12 (84.14, 88.15) | 73.87 (71.26, 76.57) | 55.94 (52.56, 59.55) | |
| AAP+ADT | 1760 | 700 | 34.52 (30.95, 37.87) | 88.04 (86.52, 89.59) | 78.24 (76.25, 80.29) | 59.37 (56.72, 62.14) | |
| DTX+ADT | 432 | 250 | 17.18 (14.10, 21.31) | 79.10 (75.31, 83.09) | 59.94 (55.25, 65.03) | 42.39 (37.43, 48.0) | |
| ADT alone | 1249 | 527 | 26.79 (25.08, 30.26) | 84.17 (82.14, 86.25) | 71.46 (68.86, 74.17) | 54.22 (50.95, 57.70) | |

AAP, abiraterone acetate plus prednisone; ADT, androgen deprivation therapy; APA, apalutamide; CI, confidence interval; DTX, docetaxel; ENZ, enzalutamide; NR, not reached

**Figure S1** Patient flow


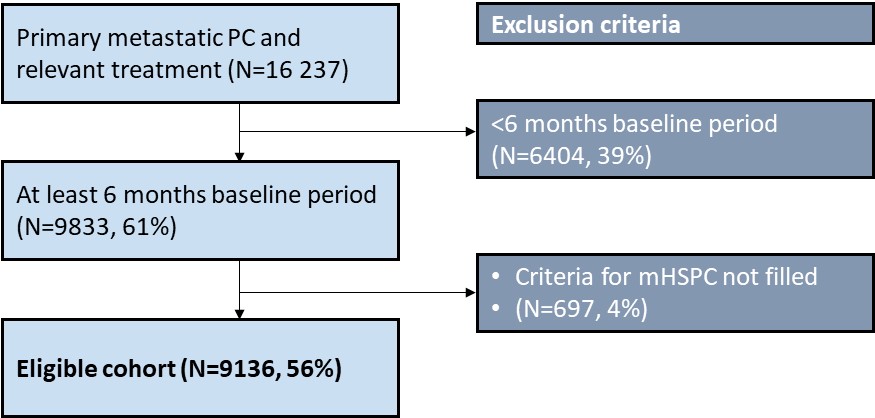


mHSPC, metastatic hormone-sensitive prostate cancer: PC, prostate cancer

Relevant treatment was defined as having completed at least 1 cycle of apalutamide, enzalutamide, abiraterone acetate plus prednisone, ADT, combined androgen blockade, docetaxel, radiotherapy, or prostatectomy.

**Figure S2** Overall survival in patients with ECOG PS 0–1, ECOG PS ≥2 and those with an unknown ECOG PS

**
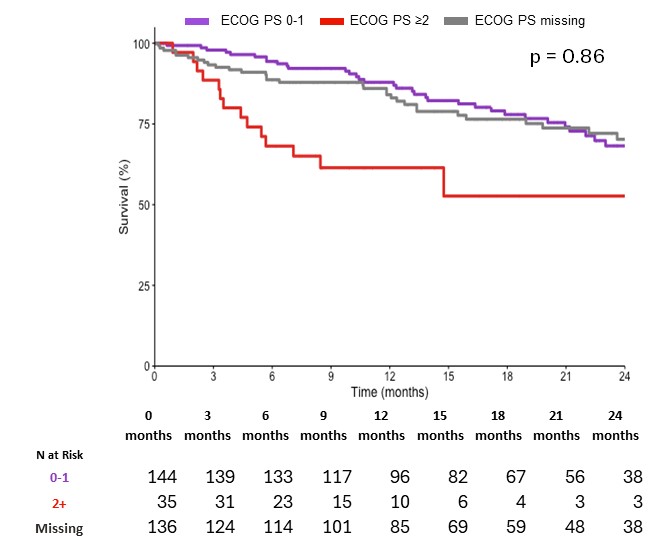
**

ECOG PS: Eastern Cooperative Oncology Group performance score

p-value comparing ECOG PS 0-1 and ECOG PS unknown

**Figure S3** Overall survival in patients with mHSPC treated with upfront DTX+ADT by number of cycles received before onset of castration resistance– Kaplan-Meier method

| **N at risk** |  |  |  |  |  |  |  |  |  |
| --- | --- | --- | --- | --- | --- | --- | --- | --- | --- |
| Group 1 | 133 | 79 | 41 | 27 | 12 | 7 | 7 | 4 | 2 |
| Group 2 | 299 | 299 | 275 | 232 | 192 | 161 | 142 | 124 | 106 |

**Group 1** received fewer than 6 cycles of DTX+ADT before onset of castration resistance.

**Group 2** received at least 6 cycles of DTX+ADT before onset of castration resistance.

ADT, androgen-deprivation therapy; DXT, docetaxel; N, number of patients
